# A Stepwise, Pilot Study of Bovine Colostrum to Supplement the First Enteral Feeding in Preterm Infants (Precolos): Study Protocol and Initial Results

**DOI:** 10.3389/fped.2017.00042

**Published:** 2017-03-03

**Authors:** Yanqi Li, Sandra M. Juhl, Xuqiang Ye, René L. Shen, Elisabeth Omolabake Iyore, Yiheng Dai, Per T. Sangild, Gorm O. Greisen

**Affiliations:** ^1^Section of Comparative Pediatrics and Nutrition, University of Copenhagen, Frederiksberg, Denmark; ^2^Department of Neonatology, Rigshospitalet, Copenhagen, Denmark; ^3^Department of Neonatology, Foshan Women’s and Children’s Hospital, Foshan, China; ^4^Department of Pediatrics and Adolescent Medicine, Rigshospitalet, Copenhagen, Denmark

**Keywords:** preterm infants, bovine colostrum, early enteral nutrition, protein intake, milk

## Abstract

**Study protocol:**

The optimal feeding for preterm infants during the first weeks is still debated, especially when mother’s own milk is lacking or limited. Intact bovine colostrum (BC) contains high amounts of protein, growth factors, and immuno-regulatory components that may benefit protein intake and gut maturation. We designed a pilot study to investigate the feasibility and tolerability of BC as the first nutrition for preterm infants. The study was designed into three phases (A, B, and C) and recruited infants with birth weights of 1,000–1,800 g (China) or gestational ages (GAs) of 27 + 0 to 32 + 6 weeks (Denmark). In phase A, three infants were recruited consecutively to receive BC as a supplement to standard feeding. In phase B, seven infants were recruited in parallel. In phase C (not yet complete), 40 infants will be randomized to BC or standard feeding. Feeding intolerance, growth, time to full enteral feeding, serious infections/NEC, plasma amino acid profile, blood biochemistry, and intestinal functions are assessed. This paper presents the study protocol and results from phases A and B.

**Results:**

Seven Danish and five Chinese infants received 22 ± 11 and 22 ± 6 ml·kg^−1^·day^−1^ BC for a mean of 7 ± 3 and 7 ± 1 days which provided 1.81 ± 0.89 and 1.83 ± 0.52 g·kg^−1^·day^−1^ protein, respectively. Growth rates until 37 weeks or discharge were in the normal range (11.8 ± 0.9 and 12.9 ± 2.7 g·kg^−1^·day^−1^ in Denmark and China, respectively). No clinical adverse effects were observed. Five infants showed a transient hypertyrosinemia on day 7 of life.

**Discussion and conclusion:**

The three-phased study design was used to proceed with caution as this is the first trial to investigate intact BC as the first feed for preterm infants. BC supplementation appeared well tolerated and resulted in high enteral protein intake. Based on the safety evaluation of phases A and B, the randomized phase C has been initiated. When complete, the Precolos trial will document whether it is feasible to use BC as a novel, bioactive milk diet for preterm infants. Our trial paves the way for a larger randomized controlled trial on using BC as the first feed for preterm infants with insufficient access to mother’s own milk.

## Introduction

Preterm birth (birth <37 weeks gestation) occurs in 10% of all pregnancies worldwide. Preterm infants suffer from various organ immaturities that may predispose them to immediate or later diseases. Adequate nutrient uptake and growth rates are required for very preterm infants to adapt well to postnatal life, but rapid enteral feeding is often difficult because the gastrointestinal tract is immature. Consequently, a suboptimal milk diet may lead to feeding intolerance, serious gastrointestinal complications (e.g., necrotizing enterocolitis, NEC), and late-onset sepsis (1).

Mother’s own milk (MM) is considered the optimal nutrition for preterm infants, and it is superior to infant formula (IF) in stimulating intestinal maturation, minimizing feeding intolerance, reducing the risk of NEC and infections, and improving long-term neurodevelopment (1). However, MM is sometimes not present in sufficient amounts or unavailable during the first days after preterm delivery. IF may be given as the first diet to supplement MM feeding, if donor milk (DM) is not available. Still, DM may remain less effective than MM to stimulate growth, secure adequate nutrient intake, and provide protection against NEC and infections (2–4). DM usually consists of pasteurized mature human milk (HM) obtained from mothers at a relatively late stage in lactation after giving birth at term. This means that DM contains a reduced amount of protein and bioactive components (5–7). DM is therefore not an ideal replacement for the first milk of the mother, the colostrum.

Bovine colostrum (BC) is the first milk from cows after birth, and we suggest that BC may be used to supplement MM during the first days of life, instead of IF or DM. BC is a rich source of protein and bioactive components, including lactoferrin, lysozyme, lactoperoxidase, immunoglobulins (Igs), as well as various growth factors (8, 9). Still, a number of nutritional characteristics of BC and milk differ from those in human colostrum (HC) and milk (e.g., levels of casein, whey proteins, oligosaccharides, and lactose). A number of phase I studies in human volunteers and phase II/III studies in patients have demonstrated a therapeutic potential of BC in gastrointestinal disorders (10). The safety and tolerability of BC has been tested in Danish pediatric patients subjected to intestinal resection (11) and chemotherapy for leukemia (NCT01766804). In a well-established piglet model of preterm infants (12), BC has repeatedly been shown to have beneficial effects in stimulating gut functions and resistance against NEC, relative to IF (13). When compared with DM and IF, BC promoted better postnatal growth, decreased diarrhea incidence, and increased brush border lactase activity in piglets (14). It is not yet clear, if the inferior quality of IF for preterm infants is due to its extensive industrial processing (e.g., heat treatment and fractionation) or suboptimal products (e.g., hydrolyzed whey proteins, vegetable carbohydrates, and vegetable oils). A general immunological hypersensitivity to bovine proteins in IF for preterm infants is unlikely, considering the overall low prevalence of cow’s milk allergy in infants (2–3%) (15).

On this background, we hypothesized that it may be feasible and justifiable to use intact BC as a supplement to MM during the first days of life, instead of DM or IF. Since BC has never been used as the first feed for preterm infants, we planned a three-phased pilot study to test the feasibility and tolerability in different clinical settings. The objectives of the pilot study were (1) to obtain the first information on whether BC used as the first nutrition is feasible and tolerable in preterm infants born between 1,000 and 1,800 g or 27 + 0 and 32 + 6 weeks’ gestational age (GA); (2) to help determine primary endpoints and sample size for a later, large randomized, controlled trial. Here, we report the entire study protocol and the results of the first two phases.

## Methods

### Study Design

**Figure 1:**
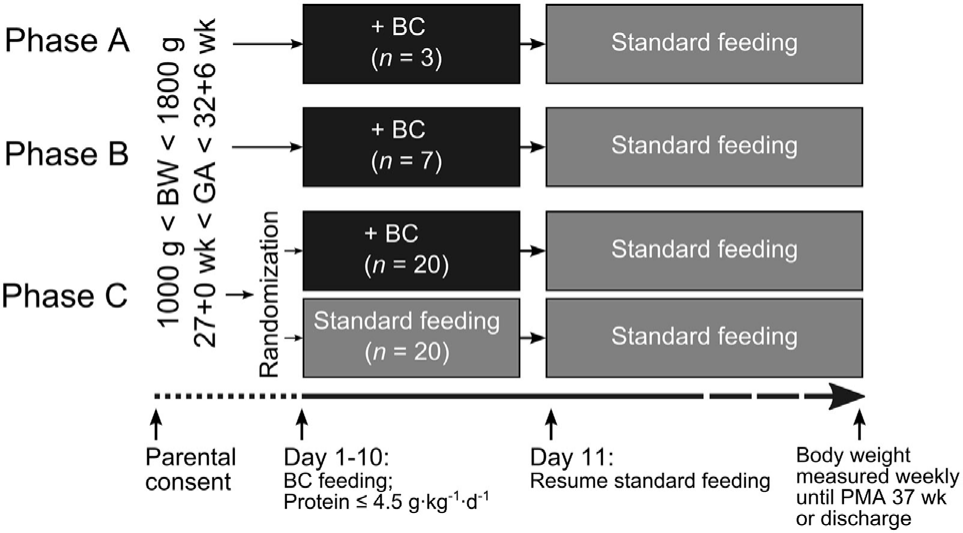
**Design of the three-phased Precolos pilot study**. BC is fed as the supplement (black boxes) when mother’s own milk is insufficient, instead of feeding donor milk (RH and HH) or infant formula (FWCH and SBMCH), according to local guidelines. In phase A, BC was given to three infants consecutively, i.e., each infant was followed until it reached full enteral feeding, and weight gain was 15 g·day^−1^ before the next infant was included. In phase B, BC was given to seven infants recruited in parallel. Phase C will be a randomized controlled trial (not yet completed). Due to differences in the normal progression of enteral feeding among hospitals, the BC intervention period was up to postnatal day 10 at RH and HH and will be up to postnatal day 14 for FWCH and SBMCH in phase C. BC, bovine colostrum; BW, birth weight; FWCH, Foshan Women’s and Children’s Hospital; GA, gestational age; HH, Hvidovre Hospital; PMA, postmenstrual age; RH, Rigshospitalet; SBMCH, Shenzhen Baoan Maternity and Child Healthcare Hospital.

Since this was the first time BC was given as the initial nutrition to preterm infants, the pilot study was designed into three phases (A, B, and C) to proceed cautiously (Figure 1). Furthermore, the study was performed in two countries with very different clinical settings to test the feasibility of giving the intervention both in a clinical setting with access to DM and early enteral feeding, and in a setting with no access to DM with later introduction of enteral feeding, representing the normal spectrum of enteral feeding practices across the world (16). In phases A and B, a stepwise, single-group design was used. Reconstituted BC was given as the first supplementary diet to MM for no longer than 10 days postnatal age. In phase A, three infants were included. Out of safety considerations, one infant was recruited and followed until he/she reached full enteral feeding and a weight gain of 15 g·day^−1^, before the next infant was recruited. Since no safety concern was raised in phase A, phase B was initiated, in which seven infants were recruited and studied in parallel. In phase C, a randomized, open labeled, and controlled trial design will be used. BC will be compared with the currently used supplementary diets to MM (IF and DM).

### Study Sites

Two Chinese hospitals, Foshan Women’s and Children’s Hospital (FWCH, Foshan) and Shenzhen Baoan Maternity and Child Healthcare Hospital (SBMCH, Shenzhen), and two Danish hospitals, Rigshospitalet (RH, Copenhagen) and Hvidovre Hospital (HH, Copenhagen) participate in this pilot study. SBMCH and HH will only include patients for the randomized phase C.

### Inclusion Criteria

Inclusion criteria differ between the countries to reflect local clinical conditions.

- FWCH and SBMCH:
  ○ Preterm infants with birth weights between 1,000 and 1,800 g
  ○ Inclusion before first feed
- RH and HH:
  ○ Preterm infants with GA between 27 + 0 and 32 + 6 wks
  ○ Inclusion before 24 h of age
- Both sites
  ○ Delivered at the study site or transferred from other hospitals within 24 h after birth
  ○ Signed parental consent

### Exclusion Criteria

- Major congenital anomalies or birth defects
- Congenital infection
- Perinatal asphyxia (umbilical or first neonatal pH ≤ 7.0)
- GA at birth <28 weeks (FWCH and SBMCH)
- Extremely SGA infant (weight SD score <−3 SD)
- Need for invasive mechanical ventilation or cardiovascular support before first BC feed

### Recruitment and Randomization

When a preterm infant was delivered, or transferred from other hospitals on the day of birth, attending neonatologists or a clinical researcher would evaluate the infant for its eligibility. If the infant fulfilled the recruitment criteria, the parent(s) would receive written and oral information about the study. Infants were included in the study after written parental consent was given.

Preterm infants were included in this study since we could not study the effect of BC as the first enteral nutrition for preterm infants by including other patient populations. Parents were informed with great care and given consideration time before inclusion. Because inclusion had to be done within a fixed period of time, this consideration time was not unlimited. If the parents found the consideration time to be too short, we accepted this and did not ask for consent.

No randomization was needed for phases A and B. For phase C, recruited infants are randomized into a control group and an intervention group in a 1:1 ratio. Random sequences of groups are generated by a computer program with randomly permuted blocks of sizes 2 and 4, written on slips, and put in opaque envelopes individually by an employee of the study sponsor. An envelope can only be opened to see the allocation after an infant has been enrolled. If multiple births are recruited together, all of them will be allocated to the same group, which will be accounted for during the data analysis. A set of 20 envelopes with random sequences will be generated for RH and HH together and coordinated by the primary investigator (PI) of RH *via* telephone call, and the recruitment will stop when 20 infants in total have been enrolled. The PI of HH will call the PI of RH when an infant is recruited and the PI of RH will open the corresponding envelope and tell which group the infant should be randomized to. For FWCH and SBMCH, two sets of 20 envelopes will be generated (one set for each) due to the difficulties related to coordination by telephone conversation. The study sponsor will coordinate and stop recruitment at the two Chinese sites when 20 infants in total have been enrolled.

### BC Intervention

The BC used in the study was prepared by mixing BC powder with cooled boiled water at room temperature (10 g powder in 50 ml water, 81kcal/100 mL). The BC powder was made from unmodified intact colostrum collected from healthy Danish dairy cows, obtained from the first and second milking after parturition. The BC was pasteurized (62.5°C for 30 min) and spray dried (ColoDan, Biofiber Damino, Gesten, Denmark). Reconstituted BC was kept in a refrigerator (4°C) for no more than 24 h. Prior to each feeding, the required amount of BC was warmed to 37°C in a water bath. The macronutrient and amino acid compositions of reconstituted BC are shown in Table 1 and are compared with that of HC, mature HM, and preterm IF.

**Table 1:** **Macronutrient and amino acid compositions of human milk (HM), human colostrum (HC), and bovine colostrum (BC), and recommendations (17) for the concentrations of nutrients in preterm infant formula**.

	HM^a^	HC^a^	BC^b^	LSRO/ASNS
Carbohydrate (g·l^−1^)	67–78	44–59	33	9.6–12.5 (g·100kcal^–^^1^)
Lipid (g·l^−1^)	32–36	20–29	35	4.4–5.7 (g·100kcal^–^^1^)
Protein (g·l^−1^)	9–12	11–32	83	2.5–3.6 (g·100kcal^–^^1^)
Whey (g·l^−1^)	6–7.2	4.3–11.1	57	
Casein (g·l^−1^)	4–4.8	3.0–5.6	26	
**Amino acids (mg·g^−1^ protein)**				
Cysteine	20	40	16	34^c^
Histidine	23	23	26	21
Isoleucine	53	36	44	52
Leucine	104	91	90	102
Lysine	71	59	73	73
Methionine	16	13	19	See cysteine
Phenylalanine	37	43	45	78^c^
Threonine	44	55	59	45
Tryptophan	ND	ND^a^	19	15
Tyrosine	46	44	54	See phenylalanine
Valine	51	54	63	53
Arginine	36	55	41	29

*LSRO, Life Sciences Research Organization; ASNS, American Society for Nutritional Sciences*.

*^a^Values are based on Ref. (9, 18, 20); ND, not determined in the reference study*.

*^b^Carbohydrate, lipid, and amino acid compositions were calculated based on the product specifications of ColoDan BC powder provided by Biofiber Damino (Gesten, Denmark). Levels of whey and casein were measured at Department of Food Science (University of Copenhagen) by Milkoscan (FOSS, Hilleroed, Denmark) before and after casein removal*.

*^c^Methionine and cysteine, and phenylalanine and tyrosine are listed in combination. The ratio of each of these combinations of amino acids should not exceed 2:1 or 1:2 without appropriate testing for adequacy*.

### Nutritional Guidelines at RH and HH (Denmark)

All recruited infants were fed according to the nutritional guidelines for preterm infants at each site. At RH and HH, the ESPGHAN nutritional recommendations are generally followed (19). Enteral nutrition was introduced as soon as possible after birth (usually within 2–6 h) and for infants of more than 28 weeks GA, volumes were increased as much as the infants tolerated. Parenteral nutrition (PN) was provided from the first day of life to infants with a BW below 1,000 g and/or GA less than 28 weeks. Older infants did generally not get any PN. Full enteral feeding was defined as 160 ml·kg^−1^·day^−1^, and PN was used until the infant received ~90% of this volume. MM was the first choice of milk and only supplemented if not available in adequate amounts. Infants were supplemented with DM in the first weeks of life, before switching to IF (Enfalac Preterm Formula; Mead Johnson Nutritionals, Nijmegen, Netherlands). Fortification (Enfamil Human Milk Fortifier, Mead Johnson Nutrition) of MM and DM normally began when infants could take 140 ml·kg^−1^·day^−1^ enterally.

#### Nutritional Guidelines at FWCH and SBMCH (China)

At FWCH and SBMCH, feeding was normally initiated within 48 h after birth with a volume of 5–10 ml·kg^−1^·day^−1^ and advanced with a rate of 10–20 ml·kg^−1^·day^−1^. The volume of full enteral feeding was defined as 120 ml·kg^−1^·day^−1^, and supplementary PN was used until infants reached this volume. When MM was absent, or not available in adequate amounts, IF (SSC 81; Abbott Laboratories, Abbott Park, IL and Enfamil A+, Mead Johnson) was used. Fortification (Similac Human Milk Fortifier, Abbott Nutrition) of MM began when the infants could take 80 ml·kg^−1^·day^−1^ enterally.

#### BC Regimen

In phases A and B, BC was given as a supplement to MM to infants instead of DM (at RH) or IF (at FWCH) until postnatal day 10. The volume of BC fed each day was determined by the expected enteral feeding volume according to the above described nutritional guidelines at each hospital and the volume of MM available. Importantly, the volume of BC intervention together with available MM was not allowed to exceed a volume equivalent to 4.5 g·kg^−1^·day^−1^ protein, e.g., the upper protein intake limit according to ESPGHAN (19). When calculating the amount of protein provided by MM, 20 g·l^−1^ was used as the estimated concentration since preterm milk usually contains a higher amount of protein than normal term milk and out of safety concerns, we did not want to risk an underestimation of the protein intake from MM (20). If the available amount of MM, and the maximal volume of BC supplementation (due to protein limitations), could not meet the expected enteral feeding volume, DM (at RH) or IF (at FWCH) was given to fulfill the needs. After the intervention period, the infants received the standard enteral diets. In phase C, 40 infants will be randomized into two groups, an intervention group and a control group. Infants in the intervention group will receive BC, as described for phases A and B, and infants in the control group will receive the respective standard diets at each site. Infants in both groups will receive standard diets after the intervention period.

### Outcome Measures

#### Demographic Characteristics of the Study Population

Major obstetrical risk factors were registered. Moreover, GA, birth weight, length and head circumference, gender, Apgar scores, inborn or transferred, days of mechanical ventilation, and days of extra oxygen were registered.

#### Clinical Outcomes

To determine the safety and tolerability of BC feeding, clinical outcomes were recorded. They included the presence of feeding intolerance (defined as at any time when feeding was withheld by the neonatologists from day 1–7 and from day 8–14), characteristics of gastric residuals, stool characteristics (all measured daily for the first 2 weeks), and any suspicion or treatment of NEC. Furthermore, bronchopulmonary dysplasia, retinopathy of prematurity, intraventricular hemorrhage, and periventricular leukomalacia diagnosed at discharge were recorded. Growth and nutritional data including weekly anthropometric measures [body weight (BWT), length, and head circumference], days to regain birth weight, days on PN, time to full enteral feeding, and nutritional intake were also recorded. In phases A and B, time to full enteral feeding was defined according to the guideline of each hospital, i.e., as the first day an infant received 160 ml·kg^−1^·day^−1^ at RH, or 120 ml·kg^−1^·day^−1^ at FWCH for a consecutive period of 72 h. Based on the experiences from phases A and B, full enteral feeding volume will be defined as 150 ml·kg^−1^·day^−1^ for all sites in phase C to have a uniform definition.

#### Paraclinical Outcomes

In phases A and B, safety measures including plasma amino acid composition, routine blood biochemistry tests, and levels of intact bovine IgG (bIgG) in plasma were measured on days 7 ± 1 and 14 ± 1. Intact bIgG molecules were quantified by electroimmunoassay (lower detection limit, 5 μg·ml^−1^) using purified bIgG as the standard and anti-bIgG as the antibody at Department of Biology, Lund University (21). In phase C, in addition to the above mentioned blood tests, a simplified three-sugar test will be performed on study day 7 ± 1 to assess intestinal function. In brief, an oral bolus containing 5% lactose, 5% lactulose, and 2% mannitol will be given to the study subjects. Blood samples will be taken at 40 min, and urine samples will be collected continuously for 6 h after administration. By analyzing the level of galactose (a digestive adduct of lactose) in blood and the ratios of lactulose/lactose and lactulose/mannitol in urine, lactose digestion, galactose uptake capacity, and intestinal permeability can be evaluated (22, 23). Fecal samples are collected on days 7 ± 1 and 14 ± 1 for fecal microbiota and short-chain fatty acid composition analyses in all three phases of the study.

### Sample Size

Fifty infants are planned to be recruited in the study: 3 in phase A, 7 in phase B, and 40 (20 + 20) in phase C. Since this study is a pilot feasibility study and no primary endpoint was set, the sample size was determined by practical considerations.

### Statistical Analysis

#### Phases A and B

Continuous variables were presented as means ± SD, and binary data were presented in counts. BUN levels measured on two different time points were compared for each hospital using paired *t*-test in Prism 5 (version 5.01, GraphPad software). Two types of growth velocity (GV) were calculated based on BWT measured weekly according to the following formulas: (1) GV (g·day^−1^) = (BWTDay *n* − BWTDay 1)/(Day *n* − Day 1) and (2) GV (g·kg^−1^·day^−1^) = [1,000 × ln(BWTDay *n*/BWTDay 1)]/(Day *n* − Day 1) (24). *Z*-score was calculated based on GA and weight using a gender specific reference (25) at birth and at 37 weeks or discharge. Day of regaining birth weight was recorded or calculated based on GV if not recorded.

#### Randomized Phase C

Outcomes from phase C will be compared for Denmark and China separately. Outcomes between BC group and the control group will be compared without knowing the group designation code. Continuous outcomes, e.g., levels of plasma amino acids, will be compared using general linear models with adjustment for potential confounders, e.g., birth weight, GA, study site, and gender. Data will be checked for normality and homoscedasticity and will be transformed when required. For binary outcomes, e.g., presence of feeding intolerance, generalized linear models will be used with adjustment for potential confounders. *p* < 0.05 is considered as statistically significant.

### Handling of Personal Data

This research project was registered at Datatilsynet (Danish Data Protection Agency). Personal data are treated with respect and will be handled according to local regulations. The outcome-related information from patient records is transferred into case report forms, if the participants’ parents give consent. Participants’ data collected during the study, including the data from the participants’ mothers, can be reviewed by the study sponsor, good clinical practice (GCP) monitor or people from the Danish authorities, who are obligated to keep the data confidential by law.

### Safety Considerations and Monitoring

Severe adverse effects (any cases of surgical NEC and death) and severe unexpected suspected adverse effects are reported to relevant ethics committees. Other severe adverse effects and several outcome measures indicating safety and tolerability of BC feeding (e.g., presence of feeding intolerance, characteristics of gastric residual, plasma amino acid composition, plasma bIgG level, and routine blood biochemistry tests) are regularly evaluated by neonatologists, clinical research staff, principal investigators at each site, as well as the study sponsor. Safety reports including above mentioned safety and tolerability data were evaluated by a data safety monitoring board (DSMB) after completion of phases A and B, respectively. DSMB consisted of three persons with expertise in neonatology, statistics, and methodology of clinical trials. Since Precolos was a pilot study, there were no defined stop rules. In case of severe unexpected, suspected adverse reactions, or other evidence of harm, termination of the study was considered by discussion between attending neonatologists, clinical research staff, principal investigators, study sponsor, and DSMB. The study sponsor ensured the GCP monitoring, according to a guideline based on the GCP guideline of International Council for Harmonization.

### Ethical Approval

The study protocol was approved by the Committees on Health Research Ethics in the Capital Region of Denmark and the Ethical Committees of Clinical Trials at FWCH and SBMCH.

## Initial Results (Phases A and B)

### Clinical Characteristics

Twelve infants were included in phases A and B; seven were from RH and five from FWCH (Table 2). The last inclusion was a set of triplets (RH) which meant that we included 12 infants in total rather than the planned 10. Mothers of three infants (two RH and one FWCH) had premature rupture of membranes >24 h. No other obstetrical complications (i.e., chorioamnionitis, placental abruption, placenta previa, preeclampsia, or eclampsia) were recorded. Apgar scores at 5 min were 10 for all infants.

**Table 2:** **Neonatal characteristics of the studied infants recruited in phases A and B at each hospital**.^a^

	RH	FWCH
*N*	7	5
GA (weeks)	29.6 ± 1.6	31.8 ± 1.6
Birth weight (g)	1,346 ± 344	1,526 ± 222
Birth *Z*-score	−0.15 ± 0.31	−0.72 ± 0.42
Birth length (cm)	38.9 ± 3.3	42.8 ± 2.4
Birth HC (cm)	27.4 ± 3.0	27.4 ± 2.4
Male gender, *n*	3	2
Antenatal steroids, *n*	5^b^	2
C-section, *n*	6	1

*C-section, cesarean section; FWCH, Foshan Women’s and Children’s Hospital, China; GA, gestational age; HC, head circumference; RH, Rigshospitalet, Denmark*.

*^a^Unless otherwise indicated, values are given as means ± SD*.

*^b^For two infants, there was no information about antenatal steroid treatment*.

### Nutrition and Clinical Outcomes at Danish Site (RH)

**Figure 2:**
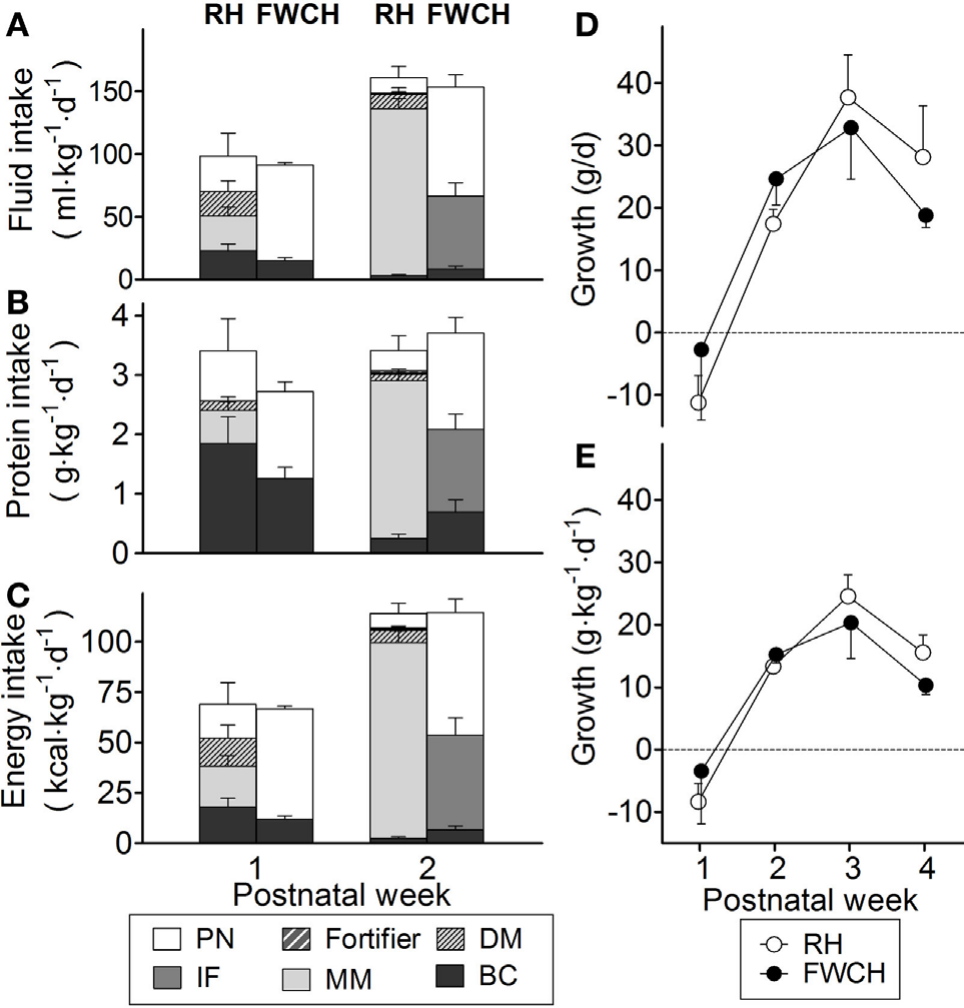
**Intake of fluid (A), protein (B), and energy (C) from different nutritional sources during the first 2 weeks of life, and growth velocity during the first 4 weeks of life, expressed as grams per day (D) and grams per kilogram per day (E)**. FWCH, Foshan Women’s and Children’s Hospital; RH, Rigshospitalet. Panels **(A–C)** show values for bovine colostrum (BC, black bars), mother’s own milk (MM, light gray bars), human donor milk (DM, light gray bars with hatching), infant formula (IF, dark gray bars), human milk fortifier (HMF, dark gray bars with hatching), and parenteral nutrition (PN, white bars). Panels **(D,E)** show values for RH (white circles) and FWCH (black circles). Values are presented as mean ± SEM for RH (*n* = 6–7) and FWCH (*n* = 4–5).

Bovine colostrum was given as the first diet on day 1 for a total of 7 ± 3 days, amounting to a total volume of 273 ± 199 ml (Table 3). During the days, BC was given, 22 ± 11 ml·kg^−1^ was fed providing 1.81 ± 0.89 g·kg^−1^ protein per day. Two infants with birth weights <1,000 g received parenteral nutrition for 11–13 days, but the remaining infants received only enteral nutrition. During the first week of life, the infants received 97 ml·kg^−1^·day^−1^ fluid, 3.4 g·kg^−1^·day^−1^ protein, and 69 kcal·kg^−1^·day^−1^. BC accounted for on average 23% of fluid, 54% of protein, and 26% of energy intakes, with the remaining deriving from MM, DM, and parenteral nutrition (Figure 2). In week 2, fluid and energy intakes increased to 161 ml·kg^−1^·day^−1^ and 114 kcal·kg^−1^·day^−1^, whereas protein intake remained the same as in week 1 (Figure 2). Time to regain birth weight was 12 ± 2 days, and time to reach full enteral feeding (160 ml·kg^−1^·day^−1^) was 22 ± 1 (Table 3). Four RH infants received 150–160 ml·kg^−1^·day^−1^ for a relatively long period. When 150 ml·kg^−1^·day^−1^ was used as the cut-off, the time was only 9 ± 3 days. GV was negative in the first week, increased throughout weeks 2–3 and decreased in week 4 (Figure 2). Feeding intolerance was observed in six infants during the first week and in one infant in week 2. Total volume of gastric residuals was 80 ± 41 ml (10.8% of enteral nutrition) in the first week (Table 3).

**Table 3:** **Outcomes of the first infants to receive BC as supplement to maternal milk in the first days of life**.^a^

	RH	FWCH
*N*	4–7	4–5
Days on PN (days)	3 ± 6	22 ± 9
BC feeding (days)	7 ± 3	7 ± 1
Total BC intake (ml)	273 ± 199	234 ± 92
TTF160 (days)	22 ± 11	ND
TTF120 (days)	8 ± 3	21 ± 8
FI in week 1, *n*	6	1
FI in week 2, *n*	1	0
GR in week 1 (ml)	80 ± 41	5 ± 5
Body weight at 37 weeks/discharge (g)	2,492 ± 326	2,138 ± 176
GA at 37 weeks/discharge (weeks)	37.3 ± 0.5	35.9 ± 0.8
*Z*-score at 37 weeks/discharge	−1.3 ± 0.7	−1.3 ± 0.6
*Z*-score change	−1.2 ± 0.6	−0.5 ± 0.5
GV (g·day^−1^)	22.0 ± 4.6	23.1 ± 5.0
GV (g·kg^−1^·day^−1^)	11.8 ± 0.9	12.9 ± 2.7
Regain birth weight (days)	12 ± 2	6 ± 2
Hypertyrosinemia on day 7, *n*	3	2
Tyrosine on day 7 (μmol·l^−1^)	171 ± 200	197 ± 163
BUN on day 7 (mmol·l^−1^)	5.8 ± 3.3	3.3 ± 1.1
BUN on day 14 (mmol·l^−1^)	2.0 ± 0.3	1.7 ± 0.6

*BC, bovine colostrum; BUN, blood urea nitrogen; FI, feeding intolerance; FWCH, Foshan Women’s and Children’s Hospital; GA, gestational age; GR, gastric residual; GV, growth velocity; HC, head circumference; PN, parenteral nutrition; RH, Rigshospitalet; TTF160, time to reach enteral feeding at 160 ml·kg^−1^·day^−1^; TTF120, time to reach enteral feeding at 120 ml·kg^−1^·day^−1^*.

*^a^Unless otherwise indicated, values are given as means ± SD*.

### Nutrition and Clinical Outcomes at Chinese Site (FWCH)

Bovine colostrum was given as the first diet on days 2–3 for a total of 7 ± 1 days, amounting to a total volume of 234 ± 92 ml (Table 3). During the period when BC was given, 22 ± 8 ml·kg^−1^ was fed providing 1.83 ± 0.52 g·kg^−1^ protein per day. BC was used as the sole enteral nutrition for the first days since MM was not available for the infants, and parenteral nutrition was used for 22 ± 9 days (Table 3). During the first week of life, infants at FWCH received 91 ml·kg^−1^·day^−1^ fluid, 2.7 g·kg^−1^·day^−1^ protein, and 67 kcal·kg^−1^ day^−1^ with BC accounting for 16, 46, and 18% of total fluid, protein, and energy intake. In week 2, fluid, protein, and energy intakes increased to 153 ml·kg^−1^·day^−1^, 3.7 g·kg^−1^·day^−1^, and −115 kcal·kg^−1^·day^−1^ (Figure 2). Time to regain birth weight was 6 ± 2 days, and time to reach full enteral feeding (120 ml·kg^−1^·day^−1^) was 22 ± 11 (Table 3). As in Denmark, GV was negative in the first week, increased throughout weeks 2–3 and decreased in week 4 (Figure 2). Feeding intolerance was observed in one infant during the first week and none in week 2. Total volume of gastric residuals was 5 ± 5 ml at FWCH (1.3% of enteral nutrition) in the first week (Table 3).

### Safety Outcomes

No clinical adverse reactions from feeding BC were observed. No infants were diagnosed with NEC, sepsis, meningitis, bronchopulmonary dysplasia, retinopathy of prematurity, and/or periventricular leukomalacia, but three (two at RH and one at FWCH) were diagnosed with grade 1 intraventricular hemorrhage. At RH, the levels of blood urea nitrogen were 5.8 ± 3.3 mmol·l^−1^ on day 7 ± 2 and decreased to 2.0 ± 0.3 mmol·l^−1^ on day 14 ± 2 (*p* < 0.05, Table 3). At FWCH, BUN levels were 3.3 ± 1.1 mmol·l^−1^ on day 7 ± 2 and decreased to 1.7 ± 0.6 mmol·l^−1^ at FWCH on day 14 ± 2 (*p* < 0.01). Amino acid levels were within normal clinical ranges, apart from five cases of hypertyrosinemia on day 7 (Table 3), that returned to normal on day 14. All levels of bIgG were below the assay detection limit.

## Discussion

Precolos is a pilot feasibility study that will provide information on feasibility, tolerability, and potential effects of BC as the first feeding for very preterm infants. Since this is the first study to feed intact BC as the initial supplemental diet for preterm infants, we designed a three-phased, stepwise study to allow close monitoring of participants for possible adverse reactions to BC feeding. The BC product used for this study was produced by gentle pasteurization and spray drying of intact BC. Milk processing, especially pasteurization, usually reduces the concentration of bioactive proteins, but pasteurized and raw BC showed similar NEC-protective effects in preterm pigs, relative to IF (26). A recent randomized, controlled trial investigated BC for preterm infants (27), but the product used (Pedimune, Merck India, Mumbai, India), contained only 23% colostrum, and the major macronutrient was carbohydrate (73%, source not specified). The product was dissolved in HM (60–67 g in 100 ml) and given to preterm infants at a dose of 2–3 ml four times a day. The study was underpowered for its endpoint but indicated that feeding this product was associated with more radiographic signs of NEC. Since a mixed product with carbohydrate as the main ingredient was used, it is difficult to compare the results with those in this trial. Previous experimental studies indicate that milk processing and addition of carbohydrate (typically vegetable-based maltodextrins) may induce NEC (28). Potentially, high osmolality may also add inflammatory damage to the immature gut (29). We suggest that mildly processed, intact BC may be a better diet for the early feeding of preterm infants. We also think that such an intervention deserves careful testing in larger clinical trials.

The first 12 preterm infants in the world have now received intact BC as a supplement to MM during the first days of life. The high level of protein in BC might be an advantage, as the same volume of BC can supply about eight times more protein than HM and three times more than IF. For safety reasons, the maximal total protein intake was set at 4.5 g·kg^−1^·day^−1^ according to the ESPGHAN guideline, and plasma amino acid and blood biochemistry values for liver and kidney functions were recorded to monitor the protein metabolism. As expected, BC provided about 50% of the protein during the first week in phases A and B, resulting in a mean intake of 3.1 g·kg^−1^·day^−1^, a level that is often difficult to reach at this early stage (30, 31). Despite this, we still observed the common drop in weight *Z*-score from day 1 until a postmenstrual age of 37 weeks or discharge in these infants (32). Plasma amino acid levels were measured to monitor potential toxicity related to high protein intake. In phases A and B, a transient hypertyrosinemia was observed in five infants on days 6–8 when tyrosine levels normally reach a peak (33). Tyrosine levels returned to normal in all infants a week later. Prolonged high levels of tyrosine may be associated with later learning disability, as indicated from studies in term infants (34). IFs with a relatively high content of casein (60–80% of total protein) may also lead to increased phenylalanine and tyrosine levels (35). The casein intake was 0.6 g·kg^−1^·day^−1^, which was much less than that in the study referred above (1.4–1.9 g·kg^−1^·day^−1^) (35). Relative concentrations of phenylalanine and tyrosine in BC were similar to those in HM, and we did not observe elevated levels of phenylalanine. Thus, the blood biochemistry results did not raise any concerns of toxicity related to excessive protein intake. After consulting with our DSMB, we decided to continue with phase C where it will be possible to compare tyrosine levels in the BC group with those in a control group.

Immunoglobulins, and especially IgGs, are abundant proteins in reconstituted BC and accounted for more than 30% of the total dietary protein in our study. For calves, colostrum provides most of the circulating IgG for passive immunity, while in human infants, this IgG is mainly transferred across the placenta before birth. We measured bIgG in plasma to check if intact bovine proteins (e.g., IgG, casein, and β-lactoglobulin) would cross the immature intestinal epithelium in preterm infants. This might sensitize the immune system and thereby predispose to NEC in preterm infants (36–38), although a direct (immunological) link between cow’s milk protein and NEC has never been documented. The prevalence of cow’s milk allergy is only 2–3% in normal infants (15) and may be even lower in preterm infants with an immature immune system. In phases A and B, we did not find any detectable levels of bIgG in plasma, probably reflecting a relatively high integrity of the intestinal epithelium in the studied preterm infants.

We decided to study BC supplementation in Denmark and in China to test its feasibility under very different conditions where access to DM was one of the differences in clinical care. The hospitals also had different conditions for determining GA, so to be cautious we used different age/weight inclusion criteria for the two sites, and we did not consider this compromising for the main aims of the study. In Denmark, mothers stayed in the wards with their infants and the infants therefore had a higher chance to receive considerable amounts of MM than those at the Chinese sites. When high amounts of MM were available, infants only received small amounts of BC, probably less than the amount required to demonstrate its potential benefits or harm. In China, fewer babies had access to MM, thereby providing better conditions to study effects/harms of BC feeding. Potentially, differences in intake of MM at the study sites need to be considered and adjusted for when analyzing the data from the later larger trials. On the other hand, it was judged important in this study to test supplementary BC feeding in different clinical settings to be able to predict the feasibility of performing a large multicenter randomized trial.

The Chinese sites and Danish sites also differed in the feeding regimens according to their use of nutritional guidelines. The Chinese sites started enteral feeding within 48 h and increased the volume slowly by 10–20 to 120 ml·kg^−1^·day^−1^ as the target full enteral volume. The Danish site normally started enteral feeding within 2–6 h after birth and increased the volume as fast as the infants could tolerate to 160 ml·kg^−1^·day^−1^ as the full enteral volume. We did not consider it possible or appropriate to standardize the feeding regimens.

In conclusion, we suggest that BC may be a relevant alternative milk diet to DM or IF as a supplement to MM in the first days of life in preterm infants. The Precolos trial is the first step to document this novel method. If Precolos can demonstrate that early BC supplementation is well tolerated, feasible, and supports one or more of the multiple outcomes to be measured in phase C, then this paves the way for a larger randomized clinical trial. Clinically relevant outcomes of such a larger trial will be intestinal functions (e.g., feeding intolerance and intestinal permeability), time to full enteral feeding, growth, length of hospital stay, NEC, and sepsis.

## Author Contributions

YL was the main study coordinator. GG was the primary investigator, and SJ was the co-primary investigator at RH. RS and EI were supportive investigators at RH. YD was the primary investigator, and XY was the co-primary investigator at FWCH. PS was the coordinating investigator and the main sponsor of the trial. YL, GG, and PS conceived and designed the study. SJ and XY were responsible for subject recruitment and data collection. YL and SJ analyzed the data and made the first draft of the manuscript. All the authors reviewed, contributed, and approved the final manuscript.

## Conflict of Interest Statement

University of Copenhagen has filed a patent application regarding the use of bovine colostrum for pediatric patients. PS is listed as the sole inventor but has declined any share of potential revenue arising from a commercial exploitation of such a patent. All other authors have no conflicts of interest.

## Abbreviations

BC, bovine colostrum; DM, donor milk; FWCH, Foshan Women’s and Children’s Hospital; GA, gestational age; HH, Hvidovre Hospital; IF, infant formula; Ig, immunoglobulin; MM, mother’s own milk; NEC, necrotizing enterocolitis; RH, Rigshospitalet; SBMCH, Shenzhen Baoan Maternity and Child Healthcare Hospital.
